# Heterologous Expression of the *AtDREB1A* Gene in Transgenic Peanut-Conferred Tolerance to Drought and Salinity Stresses

**DOI:** 10.1371/journal.pone.0110507

**Published:** 2014-12-29

**Authors:** Tanmoy Sarkar, Radhakrishnan Thankappan, Abhay Kumar, Gyan P. Mishra, Jentilal Ramjibhai Dobaria

**Affiliations:** Crop Improvement Division, Directorate of Groundnut Research, Junagadh, Gujarat, India; Institute of Genetics and Developmental Biology, Chinese Academy of Sciences, China

## Abstract

Research on genetic transformation in various crop plants using the *DREB1A* transcription factor has shown better abiotic stress tolerance in transgenic crops. The *AtDREB1A* transgenic peanut (*Arachis hypogaea* L. cv. GG 20), which was previously developed, was characterized in terms of its physio-biochemical, molecular and growth parameters. The tolerance of this transgenic peanut to drought and salinity stresses was evaluated at the seedling (18 days old) and maturity stages. Transgenic peanut lines showed improved tolerance to both stresses over wild-type, as observed by delayed and less severe wilting of leaves and by improved growth parameters that were correlated with physio-biochemical parameters such as proline content, total chlorophyll content, osmotic potential, electrolytic leakage and relative water content. The expression pattern of the *AtDREB1A* gene evaluated using qPCR at different time points demonstrated that transgene expression was induced within two hours of stress imposition. The better performance of transgenic *AtDREB1A* peanut at the seedling stage and the improved growth parameters were due to the expression of the transgene, which is a transcription factor, and the possible up-regulation of various stress-inducible, downstream genes in the signal transduction pathway under abiotic stress.

## Introduction

Peanut, or groundnut (*Arachis hypogaea* L.), is an important oil-yielding, leguminous cash crop, which is cultivated in the semi-arid tropical and sub-tropical regions of the world between 40° N and 40° S [1]. The crop is generally cultivated in low-input farming systems, primarily in the developing countries of Africa and Asia [2]. Across the world, it is cultivated over approximately 20–25 million ha in total, yielding 35–40 million tons of pods annually. India, the second largest peanut producer in the world, cultivated approximately 5–6 million ha, yielding 6–7 million tons of pods, during 2011–2012 [3], [4]. Water-deficit and soil-salinity conditions are considered to be complex abiotic stresses that affect both the growth and productivity of peanut crops [5] by disturbing the integrity of plant membrane, pigment content, osmotic adjustments, water retention capacity and photosynthetic activity [6]–[9].

Approximately 70% of the global peanut-growing areas are located in semi-arid regions, where drought is a key environmental constraint limiting peanut production. According to a recent estimate, global peanut productivity incurred an annual loss of approximately 6 million tons due to drought alone [2]. Similarly, the global salinity-affected area is approximately 830 million ha [10]. In India alone, salinity affects approximately 7.61 million ha [11], an issue that deserves major attention. Ensuring food security for the burgeoning world population is nearly impossible without considerably increasing the crop production in such marginal areas of the world [12]. It is therefore necessary to develop abiotic stress-tolerant peanut varieties that can be cultivated in the vast drought-prone and salinity-affected areas of the world [13], [14].

Because abiotic stress tolerance is a polygenic trait, developing abiotic stress-tolerant varieties through traditional breeding approaches is a difficult task. Not only that, very limited success in the genetic improvement of cultivated peanuts through conventional and marker-assisted breeding methods is also attributed mainly to the genetic isolation of the tetraploid (or amphidiploid) peanut from its wild diploid ancestors and highly conserved genome with very low polymorphism [15], [16].

Moreover, molecular breeding approaches are not widely attempted due to the difficulty in foreground and background selections. Linkage drag and only minor quantitative trait loci (QTLs) are reported for improved water use efficiency and its components [2], [17]. Ultimately, a transgenic approach could be a viable option to address this problem [18]. Globally, transgenic work for abiotic stress tolerance involves mostly the crops having cash market such as rice, maize, tomato or tobacco. Although peanut is quite critical to the livelihoods of over 650 million most food-insecure poor people living in the dryland areas of Africa and South Asia but this grain legume is not very ‘‘attractive’’ to the private sector industries [2].

The complex nature of drought and salinity responses likely involves different gene expression which could be regulated using genes encoding transcription factors (TFs) controlling gene expression under abiotic stress conditions [19], [20]. One interesting approach could be based on genetic modification of the crop of interest by introducing stress tolerance genes such as TFs, as single-action genes may not be sufficient to confer the desired abiotic stress tolerance [20]. Dehydration-responsive element-binding proteins (*DREBs*) are one of the most important classes of TFs, which are transcriptionally up-regulated during abiotic stress imposition [21]. TFs recognize a specific DNA sequence in the promoter region of targeted stress responsive genes and activate the expression of those genes [22]. The *DREBs*, which belong to the group of ethylene responsive factors (ERF), are involved in the regulation of signal transduction pathways under low temperature, salinity and dehydration conditions [8], [23]–[25].

*AtDREB1A*, a class of *DREB* from *Arabidopsis thaliana*, recognizes dehydration responsive elements/C-repeat elements (*DRE/CRT*) of the promoters of many downstream stress-inducible genes under various abiotic stresses [19], [26]–[27]. Transgenics over-expressing the *DREB* group of TFs is an efficient tool for regulating the expression of many abiotic stress-responsive genes [8], [18], [19] and it have been reported to enhance the tolerance in various crops [2], [28]–[30]. Breeding efforts to improve the drought tolerance in peanut have been undertaken by mostly focusing on improving the water use efficiency, whereas for salinity tolerance not much is reported [31]–[32].

Available reports on *AtDREB1A* transgenic peanuts have used a Spanish-type cultivar named JL 24 [5], [33]–[34], which has been phased out of cultivation in India. Here, we characterize a transgenic peanut with the *AtDREB1A* heterologous transgene, under the control of the stress-inducible *rd29A* promoter, for its tolerance to drought and salinity stresses. We utilized the Virginia Bunch variety GG20, which is high yielding, bold-seeded, and one of the most widely grown varieties in India.

## Materials and Methods

### Plant Materials

Homozygous lines of three single-copy transgenic events in the T_2_ generation (*viz*. D1, D2 and D3) of the peanut cv. GG 20, developed using *Agrobacterium tumefaciens* mediated genetic transformation [35], were used for the experiments.

### Imposition of Drought and Salinity Stresses

To evaluate tolerance to polyethylene glycol (PEG)-induced drought stress and NaCl-induced salinity stress, the physio-biochemical parameters under laboratory conditions and growth-parameters under glasshouse conditions (containment facility) were studied for both transgenic (T) and wild-type (WT) lines. The seeds were sown in a soil-sand mix (1∶1), and the 15-day-old plants were transferred to Hoagland's solution and kept in an incubation room for 3 d at 28 °C for adaptation and further growth. The experiment was conducted in three replicates and data was recorded for three plants per replication. Water deficit stress and salinity stress were created by supplementing the Hoagland's solution with 0, 10, 15, 20% PEG or 0, 100, 150 and 200 mM NaCl. The plants were grown for 7 d under water deficit stress in PEG-supplemented medium and for 12 d under salinity stress in NaCl-supplemented medium.

Following the application of stress, the roots were washed with distilled water, and the plants were transferred to Hoagland's solution (without PEG and NaCl) for recovery to normal physiological conditions. After recovery, the plants were transferred to earthen pots containing soil-sand mix (1∶1), kept in a glasshouse, and grown to maturity. During the stress treatments and recovery period in Hoagland's solution, visual observations were made to compare the severity of the wilting of leaves and the rate of recovery of the T and WT lines.

### Analysis of Growth Parameters

The growth parameters including shoot length, root length and root volume were recorded at maturity after harvesting (approximately 115–120 days after sowing). When the plants were completely dried, traits including the dry weight of pods, kernel weight, root weight, shoot weight, and total biomass were measured. The harvest index (HI) and root-shoot ratio were calculated thereafter.

### Physio-Biochemical Characterization

Physio-biochemical parameters *viz*. proline content, osmotic potential, relative water content (RWC), electrolytic leakage (EL) and total chlorophyll content were analyzed from the uppermost fully expanded leaves of seedlings that were collected prior to, during and just before removal of the stress.

#### Electrolytic leakage (EL)

EL was analyzed according to Wang et al. [36] by which fresh leaf discs (1 cm diameter) were washed with distilled water, blotted to dry and placed in 25 mL distilled water under continuous shaking for 2 h. The initial electrical conductivity (EC1) was measured using a pH/EC/TDS Meter (HI991301, HANNA, USA). The leaf discs were then boiled for 30 min in a water bath and cooled to 25°C, and the final electrical conductivity (EC2) was measured. The percent leakage of electrolytes was calculated using the formula (EC1/EC2) ×100%.

##### Relative water content (RWC)

The RWC of fresh leaf discs (1 cm dia.) from the T and WT plants was measured. First, the fresh weights (FW) of leaf disks were recorded. Next, the pre-weighed discs were floated in water in petri-plates for 8 h, after which the turgid weight (TW) of the hydrated leaf discs was recorded. The leaf discs were then dried in a hot air oven at 80°C for 72 h and weighed until a consistent dry weight (DW) was obtained. RWC was calculated using the formula RWC  =  (TW–DW)/(FW–DW) ×100, as described by Barrs and Weatherey [37].

##### Total chlorophyll content

Total chlorophyll content in the leaf tissue was determined using the dimethylsulfoxide (DMSO) method described by Hiscox and Israelstam [38]. Fifty milligrams of leaf tissue were placed in a plastic vial containing 2 mL DMSO (protected from light) and incubated in a water bath at 65°C for 12 h. Absorbance of the extract was read at 645 and 663 nm using a spectrophotometer, and the total chlorophyll content was calculated as (mg/g FW)  = 7[(20.2×OD645) + (8.02×OD663)] ×V/(1000×W), where ‘V’ is the volume of extract and ‘W’ is the weight of tissue in grams.

##### Osmotic potential

Osmotic potential was analyzed according to Bhauso et al. [4], with minor modifications. Leaf tissue samples (100 mg) of T and WT plants were first frozen in liquid nitrogen, thawed in 1.5 mL microfuge tubes at room temperature (RT) for 2 h, and then centrifuged at 12000 rpm for 10 min to collect the cell sap. The osmotic potential was estimated using a direct reading Vapor Pressure Osmometer (WESCOR, Model 5500, USA).

##### Proline content

The proline content of the leaves was estimated according to Bates et al. [39]. Leaves of WT and T seedlings (100 mg) were homogenized using a mortar and pestle with 5 mL of 3% sulfosalicylic acid and centrifuged at 5000 g for 10–15 min. The supernatant was collected and diluted to 5 mL with 3% sulfosalicylic acid. Two milliliters of glacial acetic acid and 2 mL acid ninhydrin were added to 2 mL of the supernatant and mixed well. The mixture was boiled in a water bath for 1 h and cooled. Then, 4 mL toluene was added and allowed to stand for 2–3 min for color development. The absorbance of the solution was recorded spectrophotometrically at 520 nm. A blank containing 2 mL of 3% sulfosalicylic acid without sample was also run simultaneously. The proline content was calculated according to the proline standard (100 µg/mL in 3% sulfosalicylic acid).

### Quantitative Expression of the Transgene in Transgenics Exposed to Drought and Salinity Stresses by qPCR

*AtDREB1A* gene expressions were analyzed at the transcript level in the leaf samples of all the T lines (D1, D2 and D3) exposed to a water deficit (20% PEG) and salinity stress (200 mM NaCl) for 16 h. Leaf samples were collected at 2 h intervals starting before the imposition of both salinity and drought stresses until the 16^th^ h of stress imposition. Total RNA was extracted using a Quiagen RNeasy kit, and cDNAs were prepared using a first strand cDNA synthesis kit (Fermentas, USA). Then, semi-quantitative RT-PCR was performed in triplicate with gene-specific primers (F: 5′-CCT CAG GCG GTG ATT ATA TTC C-3′, R: 5′-ACG ACC CGC CGG TTT C-3′) using a Quantifast SYBR Green PCR Kit (Qiagen, GmbH) and a step one Real-Time PCR system (Applied Biosystems California, USA). The relative quantification of *AtDREB1A* was normalized with respect to the housekeeping gene 18S rRNA as an internal control using the primers (F: 5′-GGC TCA AGC CGA TGG AAG T-3′, R: 5′AGC ACG ACA GGG TTT AAC AAG A-3′) on the Real-Time PCR system.

Comparative fold expression of the transgene was measured according to the 2^-ΔΔC^_T_ method [8], [40], and ΔC_T_ was calculated by subtracting 18S rRNA C_T_ from *AtDREB1A* C_T_ in a given sample. The ΔC_T_ value at t = 0 h, i.e., before stress imposition, was used as a calibrator. The ΔΔC_T_ value was estimated by subtracting the ΔC_T_ of the calibrator from the ΔC_T_ values at different time points. Each reaction was performed in 20 µL (volume) and consisted of 1x SYBR Green Master mix, 20 pmol of each primer and 100 ng of diluted cDNA template.

### Statistical Analysis

Statistical analysis was performed using the mean value and standard error (SE) of three replicates per analysis. Significance of a treatment effect was determined by performing a one-way ANOVA using SPSS 11.0 (Statistical Package For Social Sciences, SPSS Inc., Illinois) and a 5% probability level according to Tukey's test. The correlation coefficient was determined using PAST (PAlaeontological STatistics, ver. 1.89).

## Results and Discussion

Phenotypically, all three T lines (D1, D2 and D3) were found to be similar to WT under controlled conditions, with no growth or developmental abnormality. This indicated that the insertion of the transgene did not disrupt any major endogenous functional gene(s).

### Physio-Biochemical Characterization under Drought and Salinity Stress

#### Changes in proline content

**Figure 1:**
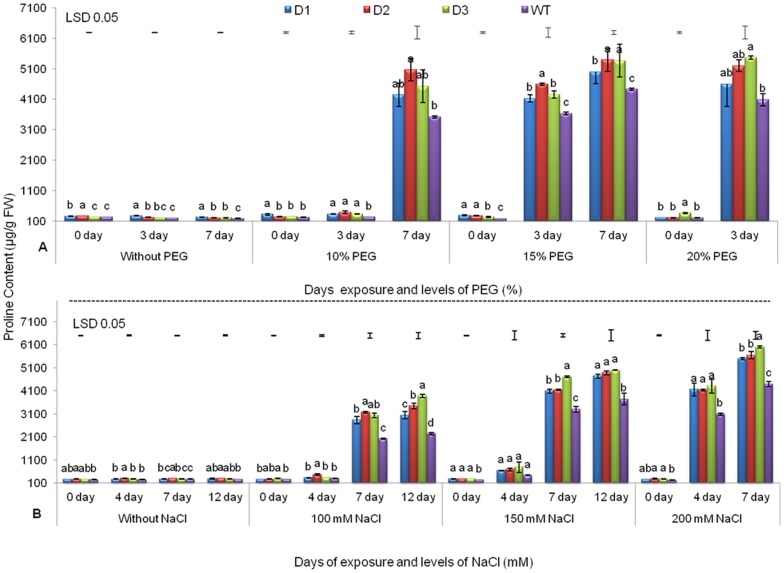
Proline Content of three transgenic lines and WT. Under various levels of PEG (A) and NaCl (B) with increasing days of stress exposure. Values represent mean activities (n = 3) ± SE at *P = *0.05. Means followed by the same lower case letters within a column are not significantly different.

Proline is known to be accumulated under water deficit and salinity stresses [5], [7], [18] and is thought to protect the plant from cellular dehydration. It is an important component of cell wall proteins, which protect membrane integrity and photosynthetic machinery [18], [41]. Under drought stress levels corresponding to 10% and 15% PEG, increases in proline accumulation were recorded on the 7^th^ and 3^rd^ d, respectively. However, at 20% PEG, all three T lines exhibited 11–25% more proline (4598–5463 µg/g FW) on the 3^rd^ d compared to WT (4011 µg/g FW) (Fig. 1A). Similar observations have been previously reported in peanut [34] and tomato [41], expressing *AtDREB1A* and *BcZAT12* genes, respectively. Similar patterns of proline accumulation were also observed in maize and tall fescue, expressing *molybdenum cofactor sulfurase* and *DREB1A/CBF3* genes, respectively, under drought stress [42], [43].

At various levels of NaCl-induced salinity stress (i.e., 100, 150 and 200 mM NaCl), proline content was relatively low up to the 4^th^ d, but drastic increases were observed on the 7^th^ d onwards. However, in T lines, at various salinity levels, significantly higher levels of proline were recorded on the 7^th^ and 12^th^ d. At 200 mM NaCl, on the 7^th^ d, all T lines exhibited 19–26% (5491 to 5997 µg/g FW) more proline accumulation than WT (4394 µg/g FW) (Fig. 1B). Significantly higher proline accumulation has also been observed under increasing salinity stress in *Populus tomentosa* and tobacco, which express *AhDREB1* and *SbSOS1* transgenes, respectively [7], [44].

Moreover, under both drought and salinity stresses, high levels of proline accumulation were recorded in T compared to WT which may be a major factor responsible for the tolerance of T peanut lines. This could be due to the up-regulation of some endogenous gene(s) by expression of the *AtDREB1A* transgene [45], [46], although further studies are required to confirm this possibility.

#### Changes in osmotic potential

**Figure 2:**
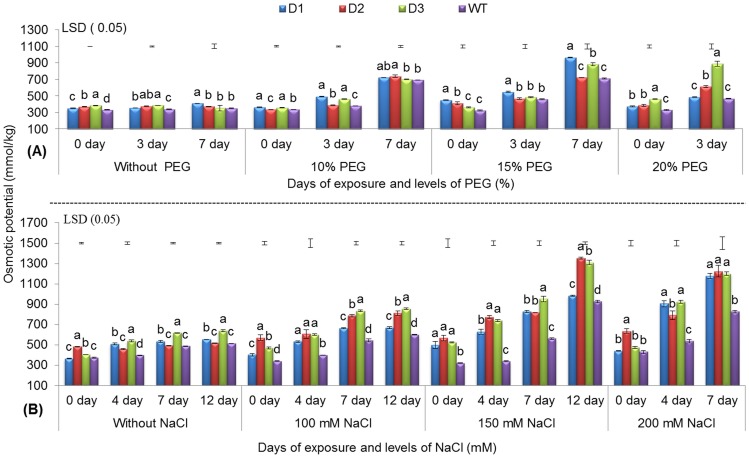
Osmotic potential of three transgenic lines and WT. Under various levels of PEG (A) and NaCl (B) with increasing days of stress exposure (refer to Fig. 1).

In general, increased osmotic potential under abiotic stress conditions confers plants with a higher water retention capacity, a lower rate of water loss, and higher water use efficiency [47]. Under drought stress, a gradual increase in osmotic potential was recorded, and T peanut lines showed better osmotic adjustment than WT during the 3^rd^ and 7^th^ d of stress imposition. In the T line D3, better osmotic adjustment was apparent, as revealed by the significantly higher osmotic potential compared with the D1 and D2 lines and with WT at 20% PEG on the 3^rd^ d (Fig. 2A). Macková et al. [48] also observed better osmotic adjustment due to the ectopic expression of the *CKX1* gene in tobacco.

For NaCl-induced salinity stress (i.e., 100, 150, and 200 mM NaCl), two of the T lines (D2 and D3) showed better osmotic adjustment than WT with increasing NaCl concentration and durations of stress exposure. Overall, the T lines exhibited 31.04 to 41.07% higher osmotic potential than WT on the 4^th^ and 7^th^ d when exposed to 200 mM NaCl (Fig. 2B). This result was supported by the observations made for *OsNHX1* and *TaSRHP* transgene containing maize [49] and *Arabidopsis* [9] respectively.

A gradual increase in osmotic potential was observed with increasing PEG and NaCl concentrations for all durations of exposure across all T and WT peanut lines. However, T lines showed better osmotic adjustment than WT under stress conditions by more efficiently increasing their osmotic potential (Fig. 2). An increase in osmotic potential is an effective mechanism adopted by plants that allows them to adapt to environmental constraints by accumulating osmolytes, such as amino acids, quaternary amines, sugar alcohols and various sugars [50].

In the present investigation, both osmotic potential and free proline content were elevated with increasing levels of PEG and NaCl-induced stresses. This supports the hypothesis that there was a positive correlation between osmotic potential and proline content at 20% PEG after the 3^rd^ d of treatment (r = 0.57, Table 1) and at 150 and 200 mM NaCl after the 7^th^ d of treatment (r>0.90, Table 2). Similar relationships were observed in tomato under water deficit stress [51]. Therefore, free proline accumulation could be one of the reasons for the enhancement in the osmotic potential of both *AtDREB1A* transgenic and WT peanut plants.

**Table 1:** Correlation coefficient (r) between the different physio-biochemical parameters under various concentration of PEG after 3 days of treatments.

	PRO10	PRO15	PRO20	OP10	OP15	OP20	RWC10	RWC15	RWC20	EC10	EC15	EC20	CHL10	CHL15
PRO15	0.79**	1.00												
PRO20	0.57*	0.54	1.00											
OP10	0.23	0.54	0.19	1.00										
OP15	0.28	0.54	0.07	0.83**	1.00									
OP20	0.32	0.54	0.57*	0.26	−0.17	1.00								
RWC10	0.48	0.54	0.19	0.83**	0.91**	−0.03	1.00							
RWC15	0.67*	0.83**	0.39	0.33	0.17	0.27	0.54	1.00						
RWC20	0.69**	0.75**	0.30	0.40	0.47	−0.04	0.75**	0.89**	1.00					
EC10	−0.24	−0.32	−0.24	−0.41	0.00	−0.57*	−0.15	−0.35	−0.05	1.00				
EC15	−0.32	−0.61*	−0.29	−0.15	0.01	−0.33	−0.32	−0.74**	−0.61*	−0.06	1.00			
EC20	−0.57*	−0.53	−0.48	−0.86**	−0.60*	−0.53	−0.77**	−0.67*	−0.59*	0.62*	0.41	1.00		
CHL10	0.46	0.47	0.59*	0.52	0.09	0.75**	0.28	0.62*	0.35	−0.58*	−0.47	−0.77	1.00	
CHL15	0.28	0.39	0.36	0.80**	0.41	0.57	0.58*	0.56	0.38	−0.80**	−0.27	−0.90	0.76**	1.00
CHL20	0.41	0.51	0.61*	0.45	0.13	0.91**	0.23	0.30	0.14	−0.42	−0.31	−0.62	0.70**	0.59*

For each parameter, average values of three *AtDREB1A* transgenic peanut lines along with WT were used. Where: PRO-proline, OP-osmotic potential, RWC-relative water content, EC-electrolytic leakage, CHL-chlorophyll content. The letters indicate the parameters followed by concentration of PEG followed by days of exposure to PEG (e.g. PRO10, Proline content at 10% PEG).* indicates a significant correlation: *P ≤0.05, and **P≤0.01.

**Table 2:** Correlation coefficient (r) between the different physio-biochemical parameters under various concentration of NaCl after 7 days of treatments.

	PRO100	PRO150	PRO200	OP100	OP150	OP200	RWC100	RWC150	RWC200	EC100	EC150	EC200	CHL100	CHL150
PRO150	0.86**	1.00												
PRO200	0.88**	0.95**	1.00											
OP100	0.86**	0.89**	0.89**	1.00										
OP150	0.82**	0.93**	0.96**	0.90**	1.00									
OP200	0.90**	0.81**	0.92**	0.81**	0.87**	1.00								
RWC100	0.54	0.82**	0.77**	0.84**	0.85**	0.59*	1.00							
RWC150	0.87**	0.93**	0.93**	0.85**	0.97**	0.89**	0.73**	1.00						
RWC200	0.64*	0.81**	0.78**	0.66*	0.89**	0.69**	0.67*	0.90**	1.00					
EC100	−0.54	−0.81*	−0.72**	−0.62*	−0.81**	−0.59	−0.74**	−0.82**	−0.90**	1.00				
EC150	−0.44	−0.75*	−0.73**	−0.51	−0.78**	−0.62	−0.68*	−0.79**	−0.83**	0.89**	1.00			
EC200	−0.57*	−0.82**	−0.83**	−0.64*	−0.89*	−0.71**	−0.77**	−0.88**	−0.89**	0.85**	0.93**	1.00		
CHL100	0.69**	0.47	0.53	0.66*	0.42	0.47	0.29	0.37	0.15	0.02	0.12	−0.09	1.00	
CHL150	0.57	0.82**	0.72	0.74	0.77**	0.56	0.76**	0.78**	0.74**	−0.82**	−0.78	−0.72	0.20	1.00
CHL200	0.62*	0.71**	0.67*	0.44	0.69**	0.62*	0.44	0.75**	0.79**	−0.73**	−0.68	−0.79	0.13	0.41

For each parameter, average values of three *AtDREB1A* transgenic peanut lines along with WT were used. The letters indicate the parameters followed by concentration of NaCl followed by days of exposure to NaCl (e.g. PRO 100, Proline content at 100mM NaCl) (Refer to Table 1).

#### Changes in RWC

Relative water content is a physiological index that is used to evaluate the water retention capacity because it acts as an appropriate parameter to measure water status and osmotic adjustments of plants under abiotic stresses [18], [52], [53]. It was observed that even under unstressed conditions, the T peanut lines exhibited significantly more RWC than WT. Moreover, with increased durations of drought stress (10, 15 and 20% PEG), a steady decline in RWC was recorded in WT compared to T plants. The WT and the T lines (D1, D2 and D3) showed 20.84, 62.60, 62.09 and 38.09% RWC (at 20% PEG), respectively, on the 3^rd^ d (S1A Fig.).

At 100 mM NaCl, with increased durations of stress, relatively smaller reductions in RWC were observed (in both T and WT). However, a sharp reduction was observed for WT compared with T at 150 mM NaCl on the 7^th^ and 12^th^ d. At higher NaCl (200 mM), on the 7^th^ d, WT and T lines (D1, D2 and D3) exhibited 38.01, 58.61, 48.28 and 61.11% RWC, respectively (S1B Fig.). Significant positive correlations between osmotic potential and RWC under 10% PEG (r = 0.83) and 150 mM NaCl (r = 0.97), were observed after the 3^rd^ d (Table 1) and 7^th^ d of treatment (Table 2). Similarly, Calcagno et al. [54] reported a close relationship between osmotic potential and RWC in *Solanum lycopersicum* under water deficit stress.

Thus, a steady reduction in RWC was recorded with increasing PEG and NaCl concentrations across T peanut lines, but the reduction was more prominent in WT. This result implies that the T lines could effectively retain more water content in their tissues than WT under increasing durations of both drought and salinity. Similar results have been reported in other transgenic crops, including maize [42], tomato [55] and tobacco [56] for drought stress and tobacco [7] and pigeonpea [57] for salinity stress.

Transgenic rice expressing the *AtDREB1A*, exhibited closure of its stomata that was correlated with its reduced water loss during transpiration under drought stress compared to WT [8]. Likewise, in our study, T lines retained more RWC, which may be due to *AtDREB1A* gene expression and its subsequent regulation of stomatal behavior during stress conditions, [8], [34] which requires further investigation.

#### Changes in electrolytic leakage

In a plant system, the cell membrane is one of the first targets of any abiotic stress, and electrolyte leakage generally reflects the membrane's stability and integrity [52]. A significant increase in electrical conductivity (EC) was recorded in WT on the 7^th^ d (at 10% and 15% PEG) compared to T lines. However, at 20% PEG, on the 3^rd^ d itself, the T lines (D1, D2, and D3) exhibited 14.37 to 32.06% less EC than WT (S2A Fig.). On a similar note, reduced EC in various T plants was observed, indicating the re-establishment of membrane integrity [54], [57].

Progressive increases in EC were noted with increased concentrations and durations of salinity exposure across all T and WT lines. On the 7^th^ d at 200 mM NaCl, WT demonstrated 61.89% EC which represents 11.23 to 38.64% higher EC than the T lines (S2B Fig.). Large negative correlations under 15% PEG (r = −0.95, Table 3) and under 200 mM NaCl (r = −0.83, Table 2) after the 7^th^ d were observed between electrolyte leakage and proline content, suggesting that proline acts as an antioxidant and helps retain membrane stability. Similarly, a significant negative correlation has been observed between proline content and electrolytic leakage in *Brassica juncea* under heat stress [58].

**Table 3:** Correlation coefficient (r) between the different physio-biochemical parameters under various concentration of PEG after 7 days of treatments.

	PRO10	PRO15	OP10	OP15	RWC10	RWC15	EC10	EC15	CHL10
PRO15	0.71**	1.00							
OP10	0.44	0.30	1.00						
OP15	0.44	0.28	0.99**	1.00					
RWC10	0.44	0.68*	0.59*	0.62**	1.00				
RWC15	0.44	0.44	0.27	0.28	0.69*	1.00			
EC10	−0.59*	−0.80**	−0.63**	−0.58*	−0.67*	−0.67*	1.00		
EC15	−0.67*	−0.95**	−0.31	−0.26	−0.54	−0.37	0.84**	1.00	
CHL10	0.57*	0.71**	0.68*	0.63*	0.45	0.35	−0.90**	−0.79	1.00
CHL15	0.42	0.62*	0.80**	0.75**	0.44	0.19	−0.80**	−0.69	0.93**

For each parameter, average values of three *AtDREB1A* transgenic peanut lines along with WT were used (Refer to Table 1).

*AtDREB1A* transgenic peanut lines showed significantly less EC compared to WT. The difference in EC between WT and T lines increased with the progression of drought and salinity stresses. Similar results were also observed in other T plants, including *BcZAT12* tomato [55], *mtlD* eggplant [52] and *VTE1* tobacco [56] under dehydration stress and *SbSOS1* tobacco [7], *MdNHX1*apple [59] and *AhCMO* cotton [60] under salinity stress.

#### Changes in total chlorophyll content

Leaf chlorophyll content directly impacts the photosynthetic rate of plants [18]. Osmotic and oxidative stresses generate extensive reactive oxygen species (ROS), which have detrimental effects on both the photosynthetic machinery and the total chlorophyll content of leaves. In addition, under drought stress (at 20% PEG), a significant decrease in chlorophyll was recorded in WT on the 3^rd^ d (S3A Fig.). Chlorophyll reduction under abiotic stress symbolizes osmotic/oxidative stress, which may have resulted from pigment photo-oxidation and chlorophyll degradation [55], [61]. Moreover it is also reported that the ectopic expression of various genes (*viz*. *Annexin*, *VTE1* and *mtlD*) helps in the retention of a greater chlorophyll content in T under dehydration stress [56], [62].

At the various salinity levels, a significant reduction in total chlorophyll in WT compared to T was observed. At 200 mM NaCl, the T line revealed 3.1–8.0% less of a reduction in total chlorophyll than WT on the 7^th^ d (S3B Fig.). It means T lines were able to retain more chlorophyll across various drought and salinity levels, which also implies its better photosynthetic capacities, resulting in improved HI. Likewise, transgenic *PeDREB2* tobacco, *mtlD* eggplant and *mtlD* peanut also showed less chlorophyll reduction under salinity stress [4], [63], [64].

Proline, which acts as a powerful scavenger of free radicals in plant metabolism, has a buffering capacity with redox potential, in addition to protecting the photosynthetic pigments [18], [41]. Thus, a significant increase in proline content in the *AtDREB1A* lines compared with WT may also be one reason for higher chlorophyll retention in T under both salinity and drought stresses (Table 3, 4), although this idea requires further confirmation.

**Table 4:** Correlation coefficient (r) between the different physio-biochemical parameters under various concentration of NaCl after 12 days of treatments.

	PRO100	PRO150	OP100	OP150	RWC100	RWC150	EC100	EC150	CHL100
PRO150	0.85**	1.00							
OP100	0.89**	0.77**	1.00						
OP150	0.85**	0.72**	0.93**	1.00					
RWC100	0.88**	0.89**	0.75**	0.65*	1.00				
RWC150	0.84**	0.90**	0.72**	0.57*	0.86**	1.00			
EC100	−0.60*	−0.60*	−0.64*	−0.36	−0.64*	−0.74**	1.00		
EC150	−0.56	−0.57	−0.42	−0.15	−0.73	−0.77**	0.81**	1.00	
CHL100	0.63*	0.50	0.66*	0.65*	0.44	0.60*	−0.37	−0.36	1.00
CHL150	0.89**	0.86**	0.77**	0.65*	0.88**	0.90**	−0.80	−0.73	0.50

For each parameter, average values of three *AtDREB1A* transgenic peanut lines along with WT were used (Refer to Table 1).

Physio-biochemical parameters, such as proline content, osmotic potential, RWC and chlorophyll content, were positively correlated, whereas electrolytic leakage was negatively correlated in T and WT peanuts under both drought and salinity stresses (Tables 1, 2).

### Variation in the Levels of the Physio-Biochemical Response

Among T- lines, significantly different levels of physio-biochemical responses were observed under both salinity and water deficit stresses, which may be due to a positional difference of the transgene integration into the T-line genome [65]–[67]. Similar types of variations have also been observed in the level of tolerance to abiotic stress, transgene expression, agronomic traits, dry-matter weight and mannitol accumulation in various T plants [4], [8], [68]. Moreover, factors such as the tissue culture regime may have conferred a positive pleiotropic effect in T peanut, and/or differential regulation of stress responsive genes under the influence of the *AtDREB1A* transcription factor may have caused the improved physio-biochemical and growth parameters, even under unstressed condition, as was observed in *AtDREB1A* transgenic rice [18].

### Quantitative Expression of the *AtDREB1A* Gene under Drought and Salinity Stress

**Figure 3:**
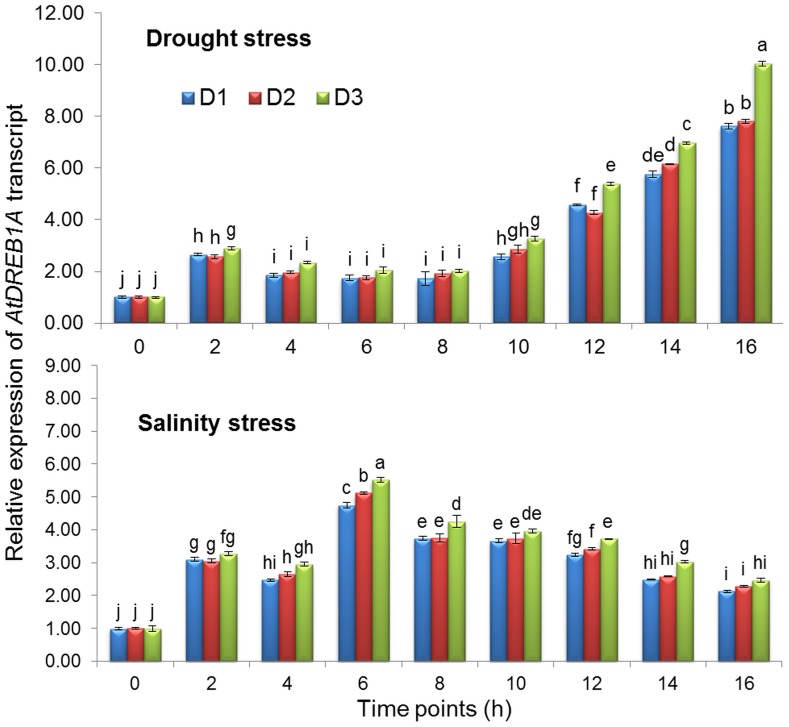
Quantitative real-time PCR analysis of expression patterns of *AtDREB1A* transgene in the leaves of transgenic lines at different time points and in response to various stresses. Values represent mean activities (n = 3) ± SD. Bars denote fold expression as compared to the expression level at 0^th^ h. Means followed by the same lower case letters within a column are not significantly different (P≤0.05).

Quantitative real-time PCR was carried out to confirm expression of the heterologous *AtDREB1A* gene in T lines (D1, D2 and D3), and differential expression of the transgene was observed at various time points during both drought and salinity stresses. Within two h of drought (20% PEG) and salinity (200 mM NaCl) stress imposition, *AtDREB1A* gene expression increased more than 2-fold (Fig. 3). However, under drought stress, consistent increases in transgene expression were recorded from the 10^th^ h, and the maximum was recorded at the 16^th^ h (>10-fold). Under salinity stress, transgene expression reached a maximum at the 6^th^ h (>5.52-fold), and a gradual declining trend was recorded subsequently until the 16^th^ h.

All the three T lines showed similar pattern of transgene expression with minute variations in 16 h treatment regime. However T line D3 showed the highest level of *AtDREB1A* transcript under both the stresses. The strong response of *AtDREB1A* gene under both the stimuli indicated that it possibly plays a role of central regulator in the signal transduction pathway that is triggered by drought and salinity stresses [9].

However, heterologous expression of the *DgWRKY3* and *ThbZIP1* genes in tobacco, under drought and salinity stresses revealed that transgene expression was more profound under drought compared to salinity stress [46], [69], whereas the reverse phenomenon was reported in *TaSRHP A. thaliana* [9]. The differential pattern of *AtDREB1A* expression in peanut, under both stresses, may be responsible for the tolerance of the T plants, characterized by less wilting and a subsequent speedy recovery, after the withdrawal of stress. Even for other crop plants, different levels of abiotic stress tolerance in T expressing various heterologous genes (*AtDREB1A*, *LbDREB*, *DgWRKY3*, *PgDREB2A* and *ThbZIP1*) were reported [24], [43], [46], [69], [70].

### Visual Observations during Drought and Salinity Stresses

**Figure 4:**
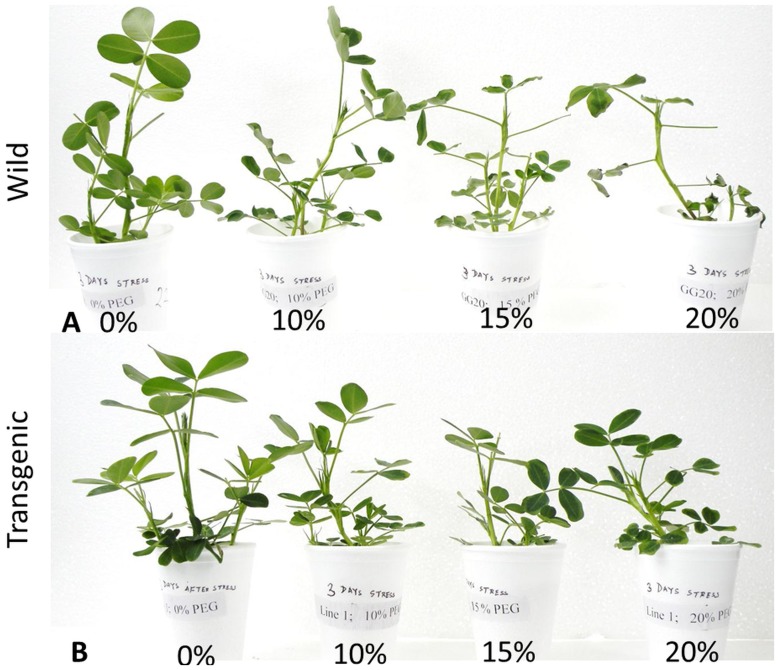
Seedlings of wild type (A) and transgenic line (B) exposed to 0, 10, 15, 20% PEG for 3 days.

During stress imposition, visual observations on wilting and its recovery rate after the withdrawal of stress were recorded for both T and WT. At 15% PEG, WT exhibited wilting on the 3^rd^ d, whereas T did not show wilting symptoms until the 5^th^ d (Fig. 4). Similarly, at 150 mM NaCl, WT and T showed wilting on the 6^th^ and 10^th^ d, respectively (S4 Fig.). Moreover, at 20% PEG and 200 mM NaCl, in WT, wilting was recorded after the 1^st^ and 3^rd^ d respectively; whereas for T wilting was observed after the 2^nd^ and 6^th^ d, respectively (Fig. 4 and S4 Fig.). The severity of wilting was more pronounced in WT, and during recovery, T recovered at a faster rate than WT (S5 Fig.).

To save the plants form further wilting and subsequent death, both stresses were withdrawn, and seedlings were recovered by transferring them to Hoagland's solution. The upper 3–4 leaves of the T plants remained either un-wilted or partially wilted. However, all leaves of the WT remained wilted, even after the 6^th^ d of stress withdrawal (20% PEG and 200 mM NaCl) (S5 Fig.). Similarly, the expression of the heterologous transgenes in tall fescue (*DREB1A*/*CBF3*), rice (*AtDREB1A*), and soybean (*AtDREB1A*) also displayed a delay in the curling of leaves under stress and a rapid recovery in T plants upon withdrawal of stress [18], [22], [43].

In our studies, even under unstressed conditions, significantly improved physio-biochemical traits were observed in the various T lines over WT (Figs. 1 and 2, S1 and S3 Figs.). Similar results have been reported for the *AtDREB1A* soybean [22] and other plant species [19], [71]. This could be due to the basal level of *AtDREB1A* expression during unstressed conditions, which is under the regulatory control of the stress-inducible *rd29A* promoter. However, upon stress imposition, there was a rapid induction and a higher level of transgene expression [22].

Furthermore, results of the RT-PCR analysis also showed a basal level of transgene expression at the transcript level under unstressed conditions (data not shown). Despite the low level of activity under unstressed conditions, the *rd29A* promoter is still considered stress-inducible and has a higher level of expression under stress compared to the *35S* promoter [26].

### Changes in Growth Parameters under Drought and Salinity Stresses

Better growth parameters of various plant species over-expressing *PgDREB2A* [24], *GsZP1* [72], *DREB1A/1B*, [8] and *mtlD* [4] genes under various abiotic stresses have been reported. For drought stress (up to 15% PEG), no significant difference in the various growth parameters was observed among T and WT (Table 5) as also reported for *AtDREB1A* soybean [23]. At 20% PEG, the three T exhibited significantly improved growth parameters, including pod weight, shoot weight, total biomass, kernel weight, root length, and shoot length, compared with WT. Moreover, under various stress conditions, an improved root-shoot ratio was recorded for T lines (Table 5) which might be because of the stress-inducible expression of *AtDREB1A* gene [33], [73]. Furthermore, in tobacco Ban et al. [70] observed more profound expression of *LbDREB* transgene in the roots compared to the leaves at 200 mM NaCl and 20% PEG-induced stresses.

**Table 5:** Growth-parameters of transgenic and WT plants under drought-stress.

Lines	PEG (%)	Growth-parameters									
		Root weight (g)	Shoot Weight (g)	Total biomass (g)	Root∶shoot ratio	Root volume (mL)	Pod weight (g)	Harvest index	Kernel weight (g)	Shoot length (cm)	Root length (cm)
D1	No PEG	4.14±0.16^a^	11.06±0.70^ab^	26.68±1.21^a^	0.37±0.01^a^	8.15± 0.36^a^	11.47±0.62^a^	0.43±0.02^a^	9.75±0.36^b^	20.94±0.88^b^	27.35±0.63^c^
D2		4.25±0.63^a^	9.66±0.27^b^	25.85±0.51^a^	0.44±0.06^a^	6.32± 0.49^b^	11.96±0.39^a^	0.46±0.02^a^	10.44±0.61^a^	21.04±0.89^b^	48.70±5.92^a^
D3		3.86±0.46^a^	12.31±0.58^a^	28.53±0.64^a^	0.32±0.04^a^	7.77± 0.20^a^	12.34±0.50^a^	0.43±0.02^a^	11.18±0.51^a^	24.87±0.40^a^	33.65±1.78^bc^
WT		3.82±0.23^a^	11.89±0.61^a^	26.75±0.91^a^	0.32±0.02^a^	7.4± 0.55^ab^	11.03±0.28^a^	0.41±0.01^a^	10.30± 0.39^a^	21.99±1.53^ab^	37.90±2.75^b^
D1	10%	3.44±0.54^a^	10.92±0.22^a^	25.4±1.50^a^	0.31±0.05^a^	6.00± 1.01^a^	11.03±0.81^a^	0.43±0.01^a^	9.71± 0.58^a^	20.37±0.70^a^	26.44±0.83^c^
D2		3.17±0.46^a^	10.66±0.37^a^	25.32±0.84^a^	0.30±0.04^a^	5.63± 0.71^a^	11.48±0.41^a^	0.45±0.02^a^	10.44±0.53^a^	20.52±0.25^a^	45.70±2.07^a^
D3		2.67±0.17^a^	11.66±0.56^a^	25.09±0.71^a^	0.23±0.02^a^	6.10± 0.32^a^	10.75±0.13^a^	0.43±0.01^a^	9.91±0.23^a^	22.79±1.74^a^	33.07±3.45^b^
WT		2.57±0.17^a^	12.26±1.13^a^	24.8±0.84^a^	0.22±0.04^a^	5.30± 0.44^a^	9.96±0.26^a^	0.40±0.02^a^	9.24±0.24^a^	21.76±1.19^a^	35.06±0.85^b^
1	15%	2.64±0.41^a^	10.64±0.35^ab^	23.06±0.42^ab^	0.25±0.04^a^	4.72± 0.81^a^	9.77±0.34^a^	0.42±0.01^a^	9.12±0.20^a^	20.12±1.86^a^	26.06±1.94^a^
D2		2.77±0.27^a^	9.82±0.60^b^	22.85±0.38^b^	0.29±0.04^a^	5.63± 0.44^a^	10.26±0.70^a^	0.45±0.02^a^	8.95±0.83^a^	19.52±0.49^a^	26.41±1.55^a^
D3		2.48±0.22^a^	11.49±0.71^ab^	24.38±0.68^a^	0.22±0.01^a^	5.61± 0.32^a^	10.40±0.47^a^	0.43±0.02^a^	9.71±0.68^a^	20.31±0.65^a^	32.34±4.11^a^
WT		2.16±0.15^a^	11.82±0.48^a^	22.99±0.14^ab^	0.18±0.02^a^	4.07± 0.26^a^	9.01±0.40^a^	0.39±0.02^a^	8.61±0.37^a^	18.74±0.42^a^	24.68±0.79^a^
D1	20%	2.51±0.39^a^	10.76±0.75^ab^	22.64±0.17^b^	0.24±0.05^ab^	4.13± 0.55^a^	9.36±0.63^a^	0.41±0.03^a^	8.43±0.48^a^	20.04±0.54^a^	25.76±3.31^b^
D2		2.88±0.34^a^	10.46±0.76^ab^	22.8±0.41^b^	0.28±0.04^a^	6.39± 0.75^a^	9.45±0.66^a^	0.41±0.03^a^	8.58±0.52^a^	19.53±0.82^a^	26.01±4.12^b^
D3		2.74±0.41^a^	12.39±0.77^a^	24.5±0.43^a^	0.22±0.04^ab^	5.57± 0.74^ab^	9.36±0.56^a^	0.38±0.03^a^	8.50±0.58^a^	20.16±0.79^a^	31.49±1.44^a^
WT		1.15±0.03^a^	8.53±0.55^b^	15.2±0.78^c^	0.13±0.01^b^	4.07± 0.38^b^	5.51±0.30^b^	0.36±0.01^a^	4.56±0.35^b^	17.39±0.74^b^	21.71±0.68^c^

The data are mean of three replicates ± SE; Means followed by the same lower case letters within a column are not significantly different (P≤0.05).

Various levels of drought stress (10 and 15% PEG) did not induce any significant adverse effect on the growth parameters in either T or WT (Table 5). However, highly detrimental effects of PEG (20%) were recorded on various growth parameters of WT compared with the T lines. Similar conclusions were reported by Jagana et al. [73] for peanut expressing the *AtDREB1A* gene under drought stress. Significantly higher root weight, root-shoot ratio and HI were observed for T across the various concentrations of salinity with increasing days of exposure (Table 6). The improvement in the growth parameters of T peanut were in agreement with the observation made by Datta et al. [8].

**Table 6:** Growth-parameters of transgenic and WT plants under salinity-stress.

Lines	NaCl	Growth-parameters									
		Root weight (g)	Shoot weight (g)	Total biomass (g)	Root: Shoot ratio	Root volume (mL)	Root length (cm)	Pod weight (g)	Harvest index	Shoot length (cm)	Kernel weight (g)
D1	No NaCl	4.76±0.31^a^	11.68±0.40^a^	29.52±0.40^a^	0.41±0.02^a^	6.67±0.43^b^	29.92±0.49^a^	13.08±0.27^a^	0.44±0.02^a^	20.39±0.56^a^	10.06±0.49^ab^
D2		3.87±0.31^ab^	10.68±1.18^a^	27.27±1.23^ab^	0.36±0.07^a^	7.83±0.55^a^	30.26±0.87^a^	12.72±0.49^a^	0.47±0.03^a^	20.97±0.87^a^	10.55±0.58^ab^
D3		4.59±0.25^a^	10.95±0.14^a^	28.87±0.34^a^	0.42±0.03^a^	6.63±0.38^b^	31.83±0.08^a^	13.33±0.30^a^	0.46±0.02^a^	21.50±0.68^a^	11.29±0.67^a^
WT		3.29±0.42^b^	10.89±0.59^a^	24.68±1.13^b^	0.30±0.03^a^	6.17±0.18^b^	30.89±0.20^a^	10.50±0.42^b^	0.42±0.01^a^	19.65±0.98^a^	9.48±0.40^b^
D1	100 mM	3.92±0.11^a^	10.57±0.37^a^	26.18±0.87^a^	0.37±0.01^a^	6.47±0.48^b^	29.43±0.78^a^	11.69±0.57^a^	0.44±0.01^ab^	20.00±1.04^a^	9.92±0.60^a^
D2		3.75±0.24^a^	10.80±0.50^a^	25.62±0.36^a^	0.35±0.02^a^	7.70±0.38^a^	30.05±0.46^a^	11.07±0.44^a^	0.43±0.02^b^	19.98±0.14^a^	10.07±0.73^a^
D3		2.81±0.19^b^	7.80±0.81^b^	20.16±1.48^b^	0.36±0.01^a^	5.53±0.43^c^	30.60±0.61^a^	9.55±0.61^b^	0.47±0.01^a^	19.87±0.99^a^	8.51±0.52^b^
WT		2.05±0.15^c^	10.95±0.44^a^	21.55±0.60^b^	0.18±0.02^b^	5.90±0.42^bc^	28.59±0.77^a^	8.55±0.51^b^	0.40±0.01^b^	19.05±0.89^a^	7.72±0.75^b^
D1	150 mM	2.86±0.23^a^	10.41±0.32^a^	23.52±0.68^a^	0.27±0.03^ab^	6.27±0.45^a^	28.14±1.07^a^	10.25±0.27^a^	0.44±0.01^a^	19.35±1.22^a^	9.31±0.72^a^
D2		2.64±0.31^a^	8.66±0.09^ab^	19.73±0.91^b^	0.30±0.05^a^	5.90±0.45^b^	29.77±1.13^ab^	8.43±0.56^b^	0.42±0.01^a^	19.70±0.74^a^	7.27±0.77^ab^
D3		2.68±0.12^a^	7.83±0.26^b^	19.24±1.37^b^	0.34±0.03^a^	4.57±0.15^c^	30.36±0.27^a^	8.73±0.49^ab^	0.45±0.01^a^	19.77±0.79^a^	8.19±0.90^a^
WT		1.76±0.31^b^	10.29±0.06^a^	18.89±0.54^b^	0.17±0.03^b^	4.13±0.12^c^	27.15±0.68^b^	6.84±0.53^b^	0.36±0.02^b^	18.82±0.67^a^	5.77±0.87^b^
D1	200 mM	2.53±0.27^a^	9.55±0.10^a^	21.18±0.62^a^	0.26±0.03^a^	5.83±0.37^a^	27.38±0.56^b^	9.10±0.41^a^	0.43±0.01^a^	19.95±0.20^a^	8.04±0.80^a^
D2		2.19±0.42^a^	8.29±0.20^b^	17.12±0.42^b^	0.26±0.05^a^	5.07±0.43^a^	27.64±0.40^b^	6.64±0.16^b^	0.39±0.01^b^	19.56±0.73^a^	5.39±0.65^b^
D3		2.25±0.20^a^	7.25±0.47^b^	17.30±1.37^b^	0.31±0.01^a^	4.57±0.79^ab^	30.17±0.27^a^	7.80±0.82^ab^	0.45±0.01^a^	19.33±0.58^a^	6.64±0.79^ab^
WT		1.18±0.04^b^	8.80±0.01^ab^	15.72±1.23^b^	0.13±0.01^b^	3.33±0.15^b^	25.73±0.40^c^	5.74±0.58^b^	0.37±0.01^b^	17.15±0.40^b^	5.04±0.52^b^

The data are mean of three replicates ± SE; Means followed by the same lower case letters within a column are not significantly different (P≤0.05).

In this study, at 20% PEG, both T and WT exhibited efficient partitioning of total biomass, with more biomass in the roots (r = 0.84) and pods (r = 0.87) than in the shoots (r = 0.78) (Table 7). Similarly, at 200 mM NaCl, more biomass was found in the roots (r = 0.75) and pods (r = 0.96) than in the shoots (r = 0.72) (Table 8). This result confirms the observations on *AtDREB1A* transgenic peanut and WT under drought stress [73].

**Table 7:** Correlation coefficient (r) between different growth- and physio-biochemical parameters under 20% PEG after 3 days of treatment.

	RW	SW	PW	TB	HI	R:S	PRO	CHL	RWC	EC
**SW**	0.48	1.00								
**PW**	0.77**	0.41	1.00							
**TB**	0.84**	0.78**	0.87**	1.00						
**HI**	0.32	−0.30	0.73**	0.31	1.00					
**R:S**	0.92**	0.12	0.72**	0.64*	0.53	1.00				
**PRO**	0.28	0.61*	0.46	0.58*	0.12	0.07	1.00			
**CHL**	0.55	0.75**	0.52	0.73**	0.02	0.31	0.61*	1.00		
**RWC**	0.65*	0.23	0.82**	0.67*	0.64*	0.69**	0.30	0.14	1.00	
**EC**	−0.65*	−0.74**	−0.76**	−0.88**	−0.25	−0.42	−0.48	−0.62*	−0.59*	1.00
**OP**	0.46	0.69**	0.43	0.64*	−0.02	0.21	0.57*	0.91**	−0.04	−0.53

For each parameter, average values of three *AtDREB1A* transgenic peanut lines along with WT were used. Where: RW-root-weight, SW-shoot-weight, PW- pod-weight, TB- total biomass, HI-harvest index, R:S-root∶shoot ratio, PRO- proline, OP-osmotic potential, RWC- relative water content, EC- electrolytic leakage, CHL-chlorophyll content. *Indicates a significant correlation: *P≤0.05, and **P≤0.01.

**Table 8:** Correlation coefficient (r) between the various growth- and physio-biochemical parameters under 200 mM NaCl after 7 days of treatment.

	RW	SW	PW	TB	HI	R:S	PRO	CHL	RWC	EC
SW	0.19	1.00								
PW	0.74**	0.56	1.00							
TB	0.75**	0.72**	0.96**	1.00						
HI	0.60*	0.15	0.87**	0.71**	1.00					
R:S	0.94**	−0.13	0.57*	0.52	0.58*	1.00				
PRO	0.61*	−0.25	0.48	0.34	0.68*	0.70**	1.00			
CHL	0.51	0.13	0.76**	0.62*	0.86**	0.46	0.67*	1.00		
RWC	0.81**	0.08	0.84**	0.72**	0.91**	0.79**	0.78**	0.80**	1.00	
EC	−0.66*	0.01	−0.72**	−0.58*	−0.86**	−0.67*	−0.83**	−0.79**	−0.89**	1.00
OP	0.63*	−0.03	0.52	0.45	0.58*	0.64*	0.92**	0.61*	0.69**	−0.71**

For each parameter, average values of three *AtDREB1A* transgenic peanut lines along with WT were used (Refer to Table 7).

Under both drought (20% PEG) and salinity stresses (200 mM NaCl), significant positive correlations were observed between RWC and the root-shoot ratio (r = 0.69 and 0.79 respectively) and between RWC and pod weight (r = 0.82 and 0.84 respectively) (Table 7 and 8). Similar relationships have also been reported between RWC and root biomass in apple [74], and between RWC and grain yield in winter wheat [75]. This reiterates the fact that improvement in the RWC has direct positive effect on the overall growth of the T plant.

Under 20% PEG, chlorophyll content was found to be positively correlated with shoot weight (r = 0.75, Table 7), indicating an accumulation of above-ground biomass. Similar relationship was established in *SAG12:ipt* transgenic *Arabidopsis* under flood conditions [76]. However, under 200 mM NaCl, chlorophyll content was positively correlated with pod weight (r = 0.76, Table 8), which indicates higher yield. Such results were also reported in *AtDREB1A* rice under drought stress [18]. In our study, T lines exhibited stress-inducible expression of the *AtDREB1A* gene, which may have led to less wilting of the leaves and the speedy recovery of seedlings. Moreover, better drought and salinity stress tolerance and improved physio-biochemical parameters in T peanut may have improved its growth performance.

## Conclusions

From this study, we can conclude that *AtDREB1A* expression improved both the drought and salinity tolerance of the T lines which could also be due to its subsequent involvement in the signal transduction pathway [18], [19], [26], [77]. The T plants showed elevated levels of proline, which resulted in better osmotic adjustments characterized by increased osmotic potential. This might be responsible for a higher water retention capacity, a lower level of ion leakage due to improved membrane integrity, and better protection of photosynthetic mechanisms. However, better retention of leaf chlorophyll content in T compared with WT could be related to higher above-ground biomass accumulation [76], which could be due to the improved rate of photosynthesis and subsequently improved productivity in T under various abiotic stresses [18].

Nevertheless, the exact mechanisms and network of *AtDREB1A*-induced regulation of native, downstream, stress-inducible genes that are responsible for improved physio-biochemical outcomes and growth parameters under various stresses are yet to be fully elucidated. Further analysis is required to determine the expression pattern of the *AtDREB1A* gene in different tissues of the T, which may reveal the reasons behind improved abiotic stress tolerance and root-shoot ratio of T over WT. Although many T peanut lines with varying degrees of improved abiotic stress tolerance have been developed around the world by numerous researchers, but to date, no commercial varieties have been developed [17]. Of the three T lines studied, D3 showed improved, combined tolerance to drought and salinity stresses, which can be used for further agronomic field trials. Subsequently, the D3 line can also be utilized in crop improvement programs as a valuable pre-breeding resource.

## Supporting Information

S1 Fig **Relative Water Content of three transgenic lines and WT.** Under various levels of PEG (A) and NaCl (B) with increasing days of stress exposure (refer to Fig. 1).
(PPT)

S2 Fig **Electrolytic Leakage of three transgenic lines and WT.** Under various levels of PEG (A) and NaCl (B) with increasing of days of stress exposure (refer to Fig. 1).
(PPT)

S3 Fig **Total Chlorophyll Content of three transgenic lines and WT.** Under various levels of PEG (A) and NaCl (B) with increasing days of stress exposure (refer to Fig. 1).
(PPT)

S4 Fig **Seedlings of wild type (WT) and transgenic (T) exposed to 0 mM (A), 100 mM (B), 150 mM (C), 200 mM (D) for 6 days.**
(PPT)

S5 Fig **Recovery of wild type (WT) and transgenic (T) lines in Hoagland's solution.** Recovery after 6 days following exposure to 20% PEG for 3 days (A) and recovery of WT and T in Hoagland's solution after 6 days following exposure to 200 mM NaCl for 7 days (B).
(PPT)
