# Supplementary material for: Heterologous Expression of the AtDREB1A Gene in Transgenic Peanut-Conferred Tolerance to Drought and Salinity Stresses
Source: PLoS One. 2014 Dec 29;9(12):e110507. doi: 10.1371/journal.pone.0110507 (PMC4278701; doi:10.1371/journal.pone.0110507)
Supplement: S3 Fig — Total Chlorophyll Content of three transgenic lines and WT. Under various levels of PEG (A) and NaCl (B) with increasing days of stress exposure (refer to Fig. 1). (PPT) [file pone.0110507.s003.ppt]

## Slide 1
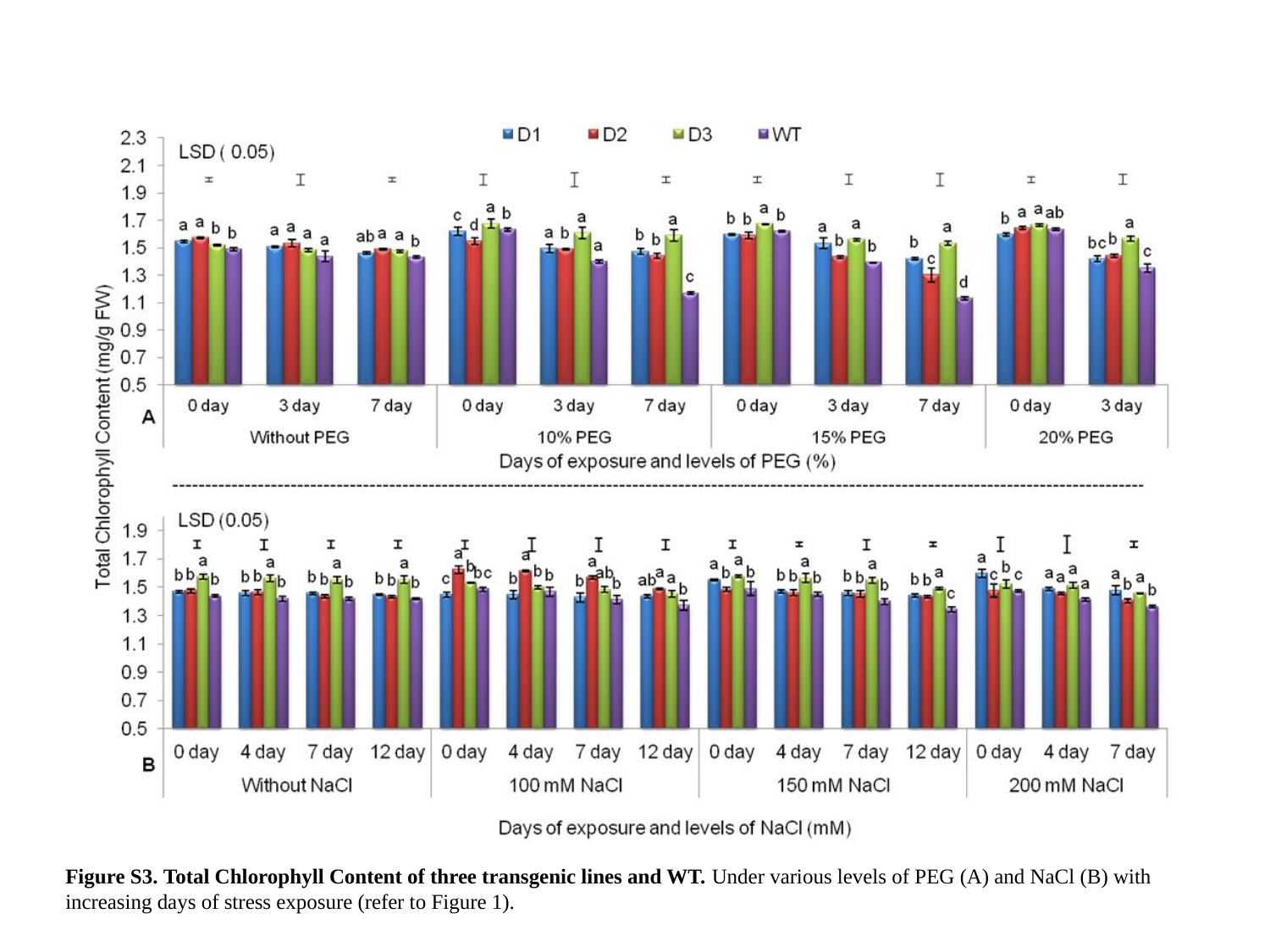

Figure S3. Total Chlorophyll Content of three transgenic lines and WT. Under various levels of PEG (A) and NaCl (B) with increasing days of stress exposure (refer to Figure 1).
