# Supplementary figures and images for: Heterologous Expression of the AtDREB1A Gene in Transgenic Peanut-Conferred Tolerance to Drought and Salinity Stresses
Source: PLoS One. 2014 Dec 29;9(12):e110507. doi: 10.1371/journal.pone.0110507 (PMC4278701; doi:10.1371/journal.pone.0110507)

## Slide 1
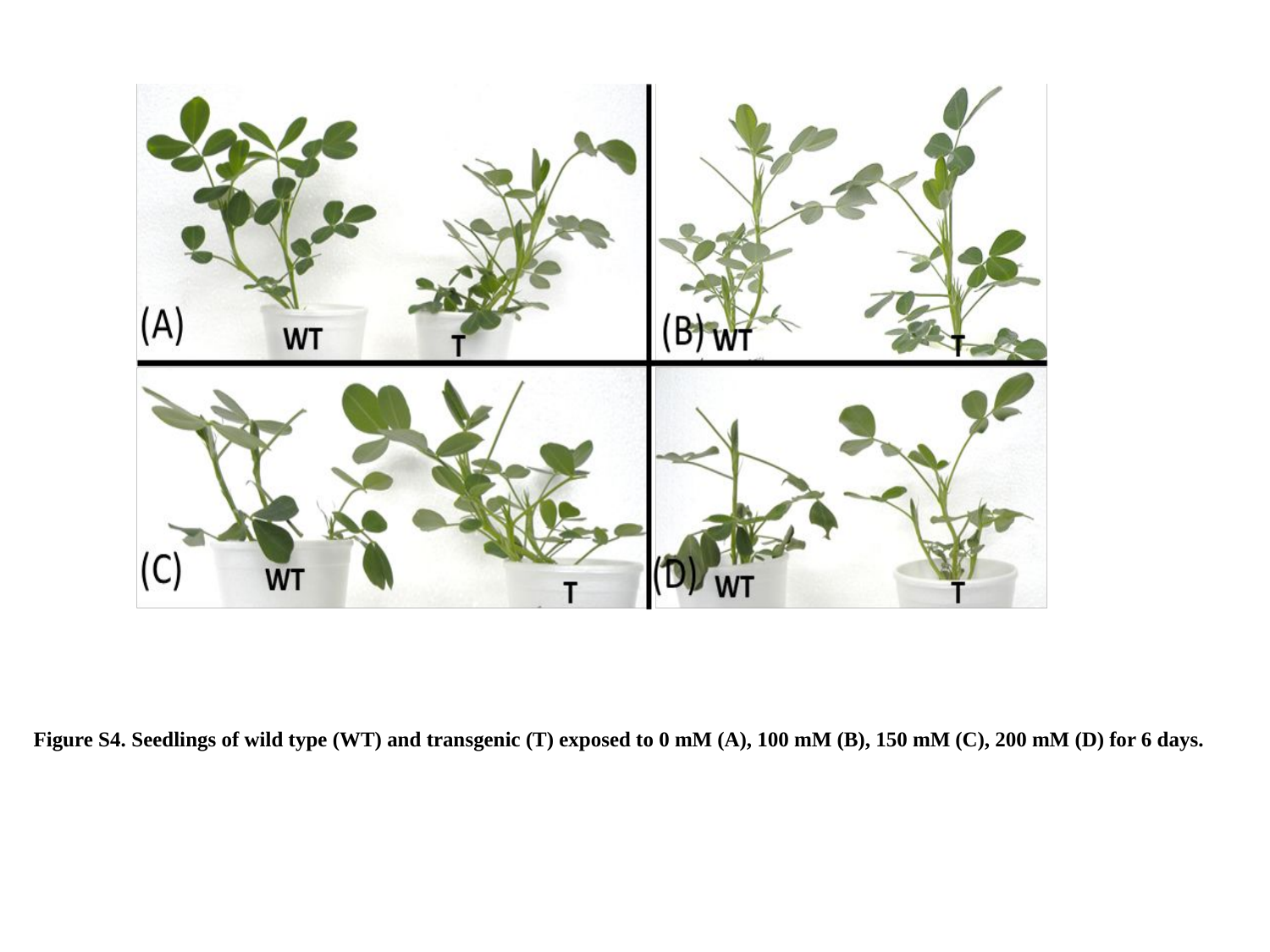

Figure S4. Seedlings of wild type (WT) and transgenic (T) exposed to 0 mM (A), 100 mM (B), 150 mM (C), 200 mM (D) for 6 days.

Supplement: S4 Fig — Seedlings of wild type (WT) and transgenic (T) exposed to 0 mM (A), 100 mM (B), 150 mM (C), 200 mM (D) for 6 days. (PPT) [file pone.0110507.s004.ppt]
