# Supplementary material for: Heterologous Expression of the AtDREB1A Gene in Transgenic Peanut-Conferred Tolerance to Drought and Salinity Stresses
Source: PLoS One. 2014 Dec 29;9(12):e110507. doi: 10.1371/journal.pone.0110507 (PMC4278701; doi:10.1371/journal.pone.0110507)
Supplement: S5 Fig — Recovery of wild type (WT) and transgenic (T) lines in Hoagland's solution. Recovery after 6 days following exposure to 20% PEG for 3 days (A) and recovery of WT and T in Hoagland's solution after 6 days following exposure to 200 mM NaCl for 7 days (B). (PPT) [file pone.0110507.s005.ppt]

## Slide 1
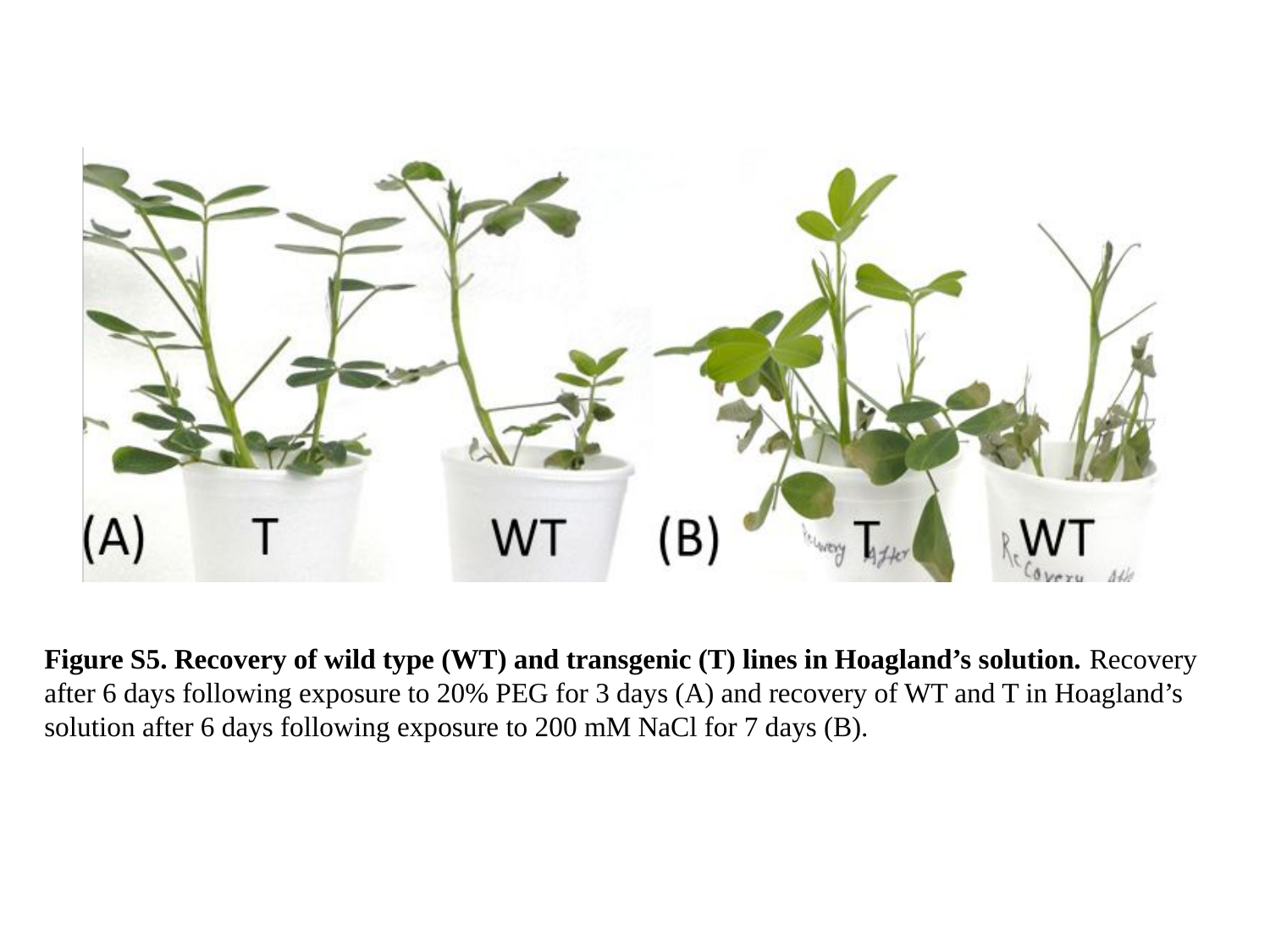

Figure S5. Recovery of wild type (WT) and transgenic (T) lines in Hoagland’s solution. Recovery after 6 days following exposure to 20% PEG for 3 days (A) and recovery of WT and T in Hoagland’s solution after 6 days following exposure to 200 mM NaCl for 7 days (B).
